# Adhesion Mechanism, Applications, and Challenges of Ocular Tissue Adhesives

**DOI:** 10.3390/ijms26020486

**Published:** 2025-01-08

**Authors:** Zuquan Hu, Xinyuan He, Lijing Teng, Xiangyu Zeng, Simian Zhu, Yu Dong, Zhu Zeng, Qiang Zheng, Xiaomin Sun

**Affiliations:** 1 Key Laboratory of Biology and Medical Engineering, School of Biology and Engineering (School of Modern Industry for Health and Medicine), Guizhou Medical University, Guiyang 550001, China; huzuquan@gmc.edu.cn (Z.H.); 2023110111219@stu.gmc.edu.cn (X.H.); ljteng@gmc.edu.cn (L.T.); zengxy@gmc.edu.cn (X.Z.); simianzhu@gmail.com (S.Z.); dongyu@gmc.edu.cn (Y.D.); zengzhu@gmc.edu.cn (Z.Z.); 2 Immune Cells and Antibody Engineering Research Center in University of Guizhou Province, Guizhou Medical University, Guiyang 550001, China; 3 Engineering Research Center of Cellular Immunotherapy of Guizhou Province, Guiyang 550001, China; 4 Engineering Research Center of Intelligent Materials and Advanced Medical Devices, School of Biology and Engineering (School of Modern Industry for Health and Medicine), Guizhou Medical University, Guiyang 550001, China

**Keywords:** medical tissue adhesive, corneal repair, wound closure

## Abstract

Corneal injury is prevalent in ophthalmology, with mild cases impacting vision and severe cases potentially resulting in permanent blindness. In clinical practice, standard treatments for corneal injury involve transplantation surgery combined with pharmacological therapy. However, surgical sutures exhibit several limitations, which can be overcome using tissue adhesives. With recent advances in biomedical materials, the use of ophthalmic tissue adhesives has expanded beyond wound closure, including tissue filling and drug delivery. Furthermore, the use of tissue adhesives has demonstrated promising outcomes in drug delivery, ophthalmic disease diagnosis, and biological scaffolds. This study briefly introduces common adhesion mechanisms and their applications in ophthalmology, aiming to increase interest in tissue adhesives and clinical ophthalmic treatment.

## 1. Introduction

Clinically, accidental trauma and postoperative damage are common. With the development of modern medicine, the limitations of traditional sutures, staplers, and medical tapes have gradually become apparent [1,2]. For example, the suturing process requires high technical skills, is time-consuming, is unsuitable for emergency situations [3], and can easily cause secondary damage and bacterial infections [4]. Staplers have poor adhesive properties for traumatized tissues and can lead to the leakage of blood and tissue fluids; moreover, they are unsuitable for tissues with low cohesiveness, such as the liver, kidneys, and lungs [5]. Therefore, new adhesive materials are urgently needed for wound closure.

Tissue adhesive, an alternative to traditional surgical sutures, has several advantages: it accelerates wound healing, prevents fluid leakage, provides noninvasive closure, is easy to operate, and requires a shorter surgical time. In brief, medical tissue adhesives are substances that can polymerize in vivo/in vitro, quickly bonding tissues in the form of sheets, powders, or crosslinked networks [6] to achieve closure and hemostasis. Typically, wound closure involves both physical adhesion and chemical adhesion, with chemical adhesion generally providing a stronger effect. The practical application is not limited to one adhesion method; various adhesion methods are used in conjunction. Initially, tissue adhesives were used solely for wound closure. With advances in modern medicine and increased demand, tissue adhesives with antibacterial properties and for adjuvant therapy and scar-free repair have been developed. The cornea, being an avascular tissue and the primary barrier of the eye, underscores the importance of developing biologically functional ocular tissue adhesives. In this study, we briefly introduce the adhesion mechanism between adhesives and corneal tissue and discuss the application directions of common tissue adhesives.

Depending on the different clinical conditions encountered in ophthalmology, tissue adhesives need to have different characteristics, such as biocompatibility, drug release ability, antibacterial potential, and injectability [7,8,9,10]. An ideal tissue adhesive should have the following properties: (1) be safe and reliable, nontoxic, noncarcinogenic, and non-mutagenic; (2) biocompatibility and noninterference with the body’s own tissue healing; (3) be suitable for wet tissue surfaces, such as blood and tears; (4) ensure rapid closure under physiological conditions; (5) have good adhesive strength and durability, with the bonded portion having a certain elasticity and toughness; (6) possessing adjustable biodegradability to adapt to the regeneration rate of different tissues; and (7) involve easy preparation and storage. In addition, tissue adhesives for ophthalmic use must maintain the normal physiological function of the eye, corneal transparency, refractive index, and patient comfort, as well as avoid the induction of scars and new blood vessel formation.

**Figure 1:**
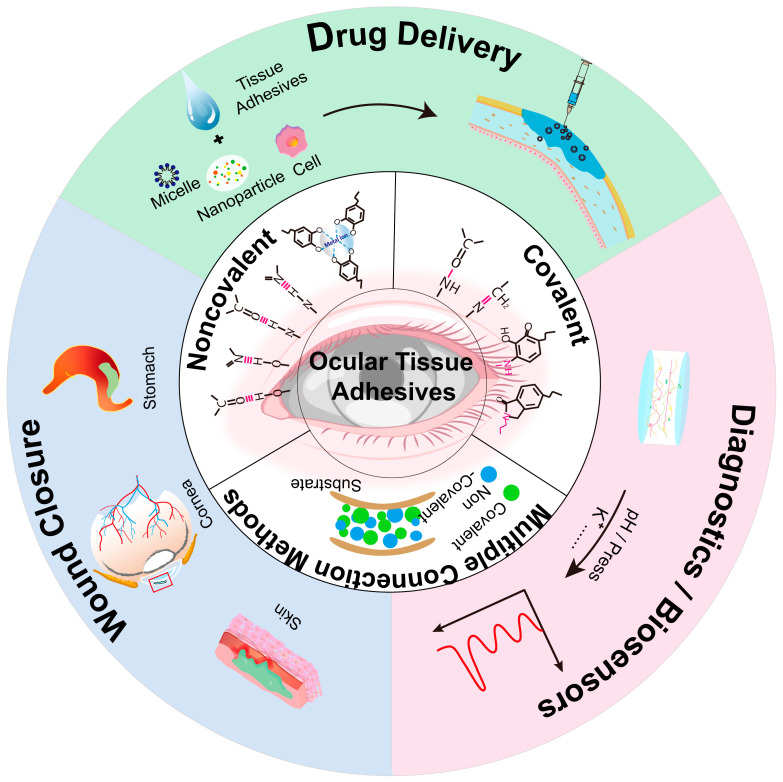
Schematic of some common ophthalmic tissue adhesives.

This study explores the fundamental principles underlying the function of tissue adhesives, their applications, and challenges in optimizing their use in medical practice. It also discusses the prospects for the next generation of ophthalmic adhesives, aiming to provide helpful insights for the readers. Figure 1 briefly illustrates the application and adhesion modes of various adhesives used in ophthalmology.

## 2. Adhesion Mechanisms Between Tissue Adhesives and Tissues

Tissue adhesives have emerged as promising alternatives to traditional suturing techniques in both surgical and clinical settings. These adhesives facilitate tissue bonding and promote healing by providing a stable environment for tissue regeneration. The basic mechanism of tissue adhesives involves interactions, such as covalent and noncovalent interactions, between the adhesive and biological tissue. These interactions can be affected by factors such as the composition of the adhesive, the surface properties of the tissue, and the physiological conditions at the site of application. Understanding these mechanisms is crucial for developing effective and biocompatible adhesives that enhance wound healing and minimize complications.

### 2.1. Van der Waals Force

The van der Waals force is a type of intermolecular force in both polar and nonpolar molecules, arising from temporary dipole moments. van der Waals forces can be categorized into several main types: (1) Dispersion Forces: present in all molecules, these forces arise from instantaneous dipoles and have the weakest bond energy, ranging from 0.1 to 5 kJ/mol; (2) Induced Forces: these occur between polar and nonpolar molecules and possess a higher bond energy, approximately 5 to 20 kJ/mol; (3) Dipole Interactions: the interactions between polar molecules are referred to as orientation forces. The strength of the van der Waals force is related to molecular polarity and molecular mass. While the bond energy between individual particles is weak, strong connections can form when many particles interact at nanoscale distances. At the hydrophobic interface, adhesion is primarily governed by van der Waals forces. van der Waals forces are used by geckos [11,12], tree frogs [13,14], insects [15], etc., to walk.

The normal corneal surface is polar [16], whereas the damaged cornea is nonpolar, leading to various van der Waals interactions, which adhesives can exploit.

### 2.2. Hydrogen Bond

Compared to van der Waals forces, a hydrogen bond is a special intermolecular force that forms under more stringent conditions; therefore, the bond energy is slightly stronger, typically ranging from 15 to 40 kcal/mol. In addition to the common hydrogen bonds found in water, ammonia compounds, and inorganic acids, hydrogen bonds can be unique. The DNA double-helix structure proposed in 1953 highlights hydrogen bonds as crucial connectors between DNA strands, with slight variations in bonding between different base pairs: A-T pairs require two hydrogen bonds (K_assoc_ ≈ 10^2^ M^−1^ in chloroform) and G-C pairs require three hydrogen bonds (K_assoc_ ≈ 10^3^–10^5^ M^−1^), resulting in stronger interactions [17]. The association constant (K_assoc_) is a parameter used to quantify the strength of interactions between molecules. It represents the equilibrium ratio of the concentration of bound complexes to the concentrations of free binding partners.

**Figure 2:**
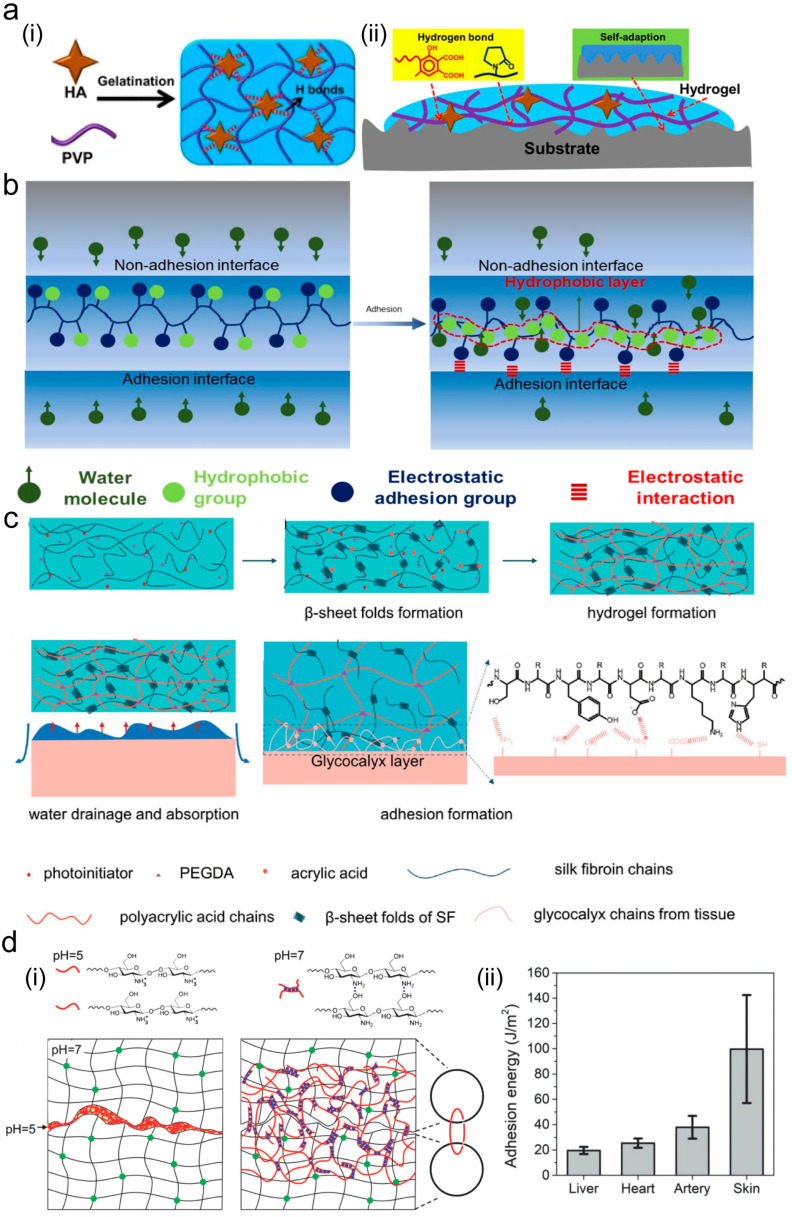
Physical adhesion mechanism. (**a**) Mechanism of hydrogen bonding (**i**) and tissue adhesion (**ii**) (Reprinted with permission from Ref. [19]. 2022, Frontiers); (**b**) mechanism of electrostatic interaction adhesives (Reprinted with permission from Ref. [20]. 2022, ACS Publications); (**c**) topological entanglement between gel and tissue in short range (Reprinted with permission from Ref. [21]. 2022, Wiley); and (**d**) mechanism of gelation (**i**) and detection of adhesion strength (**ii**) in topological networks (Reprinted with permission from Ref. [22]. 2018, Wiley).

Hydrogen bonds are crucial for the formation and adhesion of tissue adhesives [18]. Yu et al. [19] created a humic acid (HA)/polyvinylpyrrolidone (PVP) complex hydrogel with self-healing and adhesion properties. They utilized the hydrogen bond between humic acid and polyvinylpyrrolidone to construct the hydrogel (Figure 2a(i)) while enhancing interface interactions through hydrogen bonds between the hydrogels and the functional groups of the substrates (Figure 2a(ii)). This type of gel with hydrogen bonds as the main adhesion mode offers the advantage of repeated use.

### 2.3. Electrostatic Interaction

Electrostatic interaction is one of the most widely used noncovalent interactions. In humid environments, many biological surfaces carry positive or negative charges, leading to attractive or repulsive forces upon contact. Electrostatic interactions are particularly prevalent in nanoparticles, macromolecules, and liquid environments [23]. These interactions are also found in the adhesive cells of Cnidaria and the extracellular polymeric matrix (EPM) of bacteria [24].

The strength of electrostatic force depends on the motion and positioning of the particles [2]. The adhesion strength resulting from electrostatic interactions is relatively weak (approximately 10 kPa) and exhibits extremely poor stability [25,26,27]. Tian et al. [20] developed an electrostatic adhesive (PAGMA-1) that is capable of effectively bonding wet dynamic tissue to the electrode material of sensors. When applied to a moist surface, gelatin absorbs water, exposing the sulfonic acid groups of 2-acrylamido-2-methylpropane sulfonic acid and rapidly adhering to the tissue through electrostatic interactions (Figure 2b). The absorbed water causes the hydrophobic groups to cluster, forming an internal hydrophobic layer that reduces water diffusion at the adhesion interface. This method achieved an adhesion strength of 85 kPa; moreover, it eliminated the interference of small numbers of water molecules at the interface, ensuring strong adhesion. Roy et al. [28] designed an adhesive that uses the interaction of positive and negative charges to achieve adhesion between gels and tissue. A 10 mm incision was made on porcine liver tissue, and gels with different charges were used for adhesion. The neutral gel and negatively charged gel adhered successfully; however, the negatively charged gel slipped off after a few seconds, indicating insufficient adhesion with the tissue. Additionally, the adhesion between gels, pork tissues, and glass plates with different charges is more stable than that between gels and tissues, with no shedding observed.

### 2.4. Topological Structure

Topological adhesion requires two objects with mutually adhered polymer networks, without the need for functional groups for chemical coupling [6]. The adhesive solution is distributed and penetrates into the tissue. Under specific conditions (pH, temperature, and ions), an interlocking network is triggered to form upon which a topological entanglement between the two objects is created [29]. Liu et al. [21] developed a strong adhesive by leveraging a double-network structure of fibroin protein and polyacrylic acid. Short-range interface topological adhesion was achieved through the adsorption of tissue fluid by the hydrogel during adhesion (Figure 2c). With the assistance of hydrogen bonding and electrostatic interactions, the adhesive strength of the gel can reach 1040 J/m^2^, with a strength of 900 J/m^2^ on wet tissue surfaces. This adhesive has shown promising results in in vitro experiments for gastric perforation and pneumothorax closure. Leveraging chitosan’s pH sensitivity, Yang et al. [22] added a chitosan solution with a pH of 5 at the interface of two hydrogels with a pH of 7, creating a new entanglement network between two hydrogels (Figure 2d). They utilized this chitosan adhesive to bond polyacrylamide hydrogels with different soft organs, including the liver, heart, artery, and skin, demonstrating relatively high toughness. Figure 2d(ii) demonstrates that the adhesive strength can reach 20 J/m^2^ on liver tissue and 100 J/m^2^ on the skin surface.

### 2.5. Covalent Bonding

Covalent bonding is an important means of bonding in the preparation of tissue adhesives. Covalent bond strength ranges from 100 to 1000 kJ/mol [30], far exceeding that of physical connections [31,32]. When the adhesive contacts the tissue, the functional groups at the two interfaces interact, forming corresponding chemical bonds, such as imine bonds, borate ester bonds, and metal–phenol coordination bonds, ensuring robust tissue adhesion. In studies of wound closure, most adhesion between materials and tissues is achieved through covalent bonds. Common functional groups include primary amines, aldehydes, isocyanates, carboxylic acids, and phenolic hydroxyls, which typically react with primary amines, carboxylic acids, thiols, or hydroxyls in tissues [33]. Zhang et al. [34] developed functionally coupled PEG–lysozyme (PEG-LZ) hydrogels for antibacterial purposes. By connecting the -NHS group in PEG-NHS and the -NH_2_ group in the lysozyme, a strong connection is formed. In addition, the residual -NHS reacts with the amino group in the tissue to form a covalent ester bond, which realizes tissue adhesion and connection at the same time. However, this type of adhesive cannot fully conform to the corneal wound, because it reacts gradually upon contacting the tissue without penetrating deeply.

**Figure 3:**
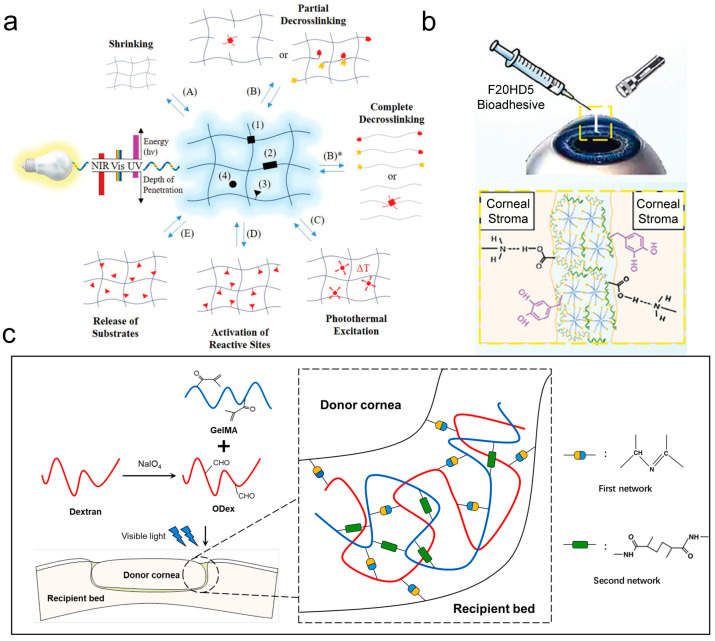
Photocuring connection. (**a**) Mechanism of photoresponsive hydrogel. Black stands for photoresponsive groups, which at various locations: (1) at crosslinking points, (2) along the polymer or supramolecular backbone, (3) on the side chains, or (4) dissolved within the aqueous medium of hydrogels. Depending on their specific location and type, photoresponses may include shrinking (A) and partial de-crosslinking (B), which typically lead to an increase in water uptake and hydrogel volume. Conversely, a photoinduced enhancement in crosslinking generally results in hydrogel contraction. Complete de-crosslinking ultimately leads to hydrogel degradation (B)*. Additional responses encompass: (C) photothermal excitation, (D) activation or deactivation of reactive sites, and (E) substrate release or capture (Reprinted with permission from Ref. [37]. 2019, Wiley); (**b**) interaction mechanism between light-cured gel and ocular surface (Reprinted with permission from Ref. [38]. 2023, Wiley); (**c**) interaction mechanism of photoresponsive double-network adhesive, the blue line represents the main body of GelMA, while the red line indicates ODex (Reprinted with permission from Ref. [39]. 2021, ScienceDirect).

To fit the corneal defect better and assist adhesion, light curing is typically used to prepare ophthalmic tissue adhesives [35,36]. As shown in Figure 3a [37], photoresponsive molecules may exist at the crosslinking points, supramolecular backbones, side chains, or dissolved in the interstitial space of the hydrogel. The light response can be categorized into contraction, partial decrosslinking, and complete decrosslinking, with the latter leading to hydrogel degradation (B)*. Additional responses include photothermal excitation, activation or inactivation of active sites, and the release or capture of substrates.

Wang et al. [38] employed temperature-sensitive F127DA to improve adhesion at the ocular surface, while a photocuring reaction further strengthened the bond between the adhesive and the tissue (Figure 3b). To further increase adhesion, Zhao et al. [39] combined Gelatin Methacryloyl (GelMA) with oxidized dextran (ODex), which can photopolymerize in situ and result in a smooth, firm surface after “suturing” which meets physiological requirements. Figure 3c shows that the first Schiff base network forms an initial gel by crosslinking the amino groups on the corneal collagen with the aldehyde groups in the adhesive, facilitating tissue adhesion. The second network involves a free radical polymerization reaction that enhances the internal crosslinking in GelMA, resulting in a more compact gel structure. This dual structure provides strong adhesion, enabling the gel to solidify within 4 min and withstand pressures of >285 mmHg, well above normal intraocular pressure.

**Figure 4:**
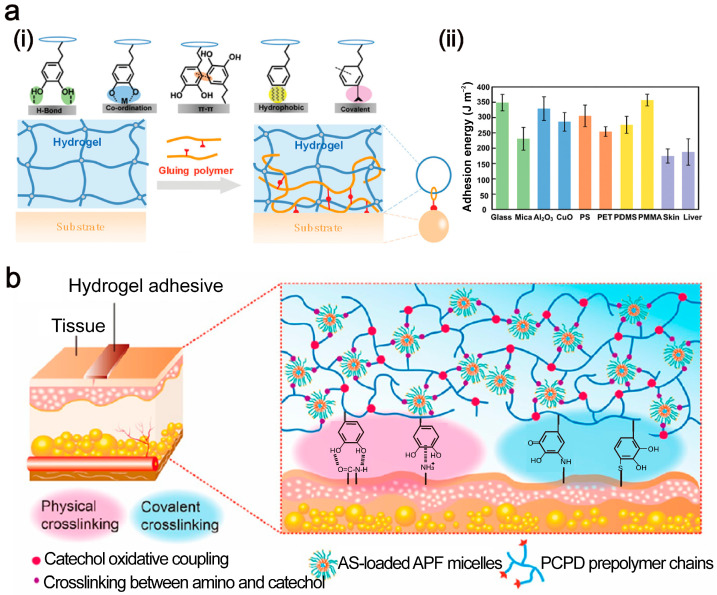
Adhesion methods of catechol groups. (**a**) Adhesion modes (**i**) and adhesion strength (**ii**) between stitch-bonding adhesive and different materials (Reprinted with permission from Ref. [41]. 2020, Wiley); (**b**) schematic of adhesion between catechol groups and tissues (Reprinted with permission from Ref. [42]. 2023, ACS Publications).

Because the eye surface is moist with tears, the adhesive must overcome the wet tissue environment and maintain stable adhesion. The adhesion mode of mussels has indicated that the catechol group is an effective functional group for underwater adhesion [40]. Gao et al. [41] designed a stitch-bonding strategy for wet tissue adhesion by integrating various functional groups and polymer chains into an adhesive to ensure stable underwater adhesion. The prepared hydrogel interacts with sodium periodate through a catechol-linked macromolecular chain and forms an interpenetrating network through the coupling of two catechol units. Numerous residual catechol groups are involved in interactions such as hydrogen bonding, π–π stacking, and metal coordination. Oxidized catechol can also react with active amine or thiol groups in tissues through Michael addition (Figure 4a(i)). The mercaptan group acts as a nucleophile, attacking the β-carbon of α, β-unsaturated carbonyl compounds to form a new carbon–sulfur bond. This is followed by a proton transfer, leading to the formation of the final mercaptan derivative. As shown in Figure 4a(ii), the hydrogel adheres to substrates such as glass, CuO, and PET, achieving an adhesion strength of 300 J/m^2^, whereas upon adhering to tissues, such as the skin and liver, the adhesion strength reaches 150 J/m^2^. Zhao et al. [42] used catechol groups in an adhesive along with free amino and sulfhydryl groups at the tissue interface to create stable adhesion (Figure 4b).

### 2.6. Other Adhesion Mechanisms

Traditional tissue adhesives are difficult to reuse because the covalent bonds break irreversibly. This has prompted the development of a new type of covalent bond called the dynamic covalent bond, which can undergo reversible exchange under specific conditions. Such bonds can reversibly form and break by modulating factors such as pH [43], pKa (acidity coefficient), or external environmental conditions. The Schiff base bond is a typical dynamic covalent bond. Reyes et al. [44] effectively sealed rabbit corneal incisions using a chondroitin sulfate-aldehyde adhesive, where the aldehyde group reacted with the abundant amino groups in the tissue through a typical Schiff base reaction.

Adhesives usually bond tissues through covalent connections, resulting in a high adhesion strength but damaging the biocompatibility of adhesives [25,45,46]. A single adhesion method is usually weak, and there are many ways to combine methods together in practical applications. Li et al. [46] developed a heat-triggered reversible adhesive which uses temperature change to control adhesion or detachment. Tannic acid was used to pretreat the tissue, followed by the application of temperature-sensitive materials. This double-layer structure includes multiple bonding methods, such as hydrogen bonding, electrostatic interactions, and covalent bonding, enhancing the connection. Table 1 presents the common adhesive types, detailing their composition, adhesion strength, applications, etc.

**Table 1:** Introduction of common adhesives.

Components	Binding Mechanism	Adhesion Strength	Application	Ref.
Gelatin, HA	Schiff base reaction	13 kPa	Corneal reconstruction	[47]
Fibrin	NA	4 kPa	NA	[47]
Chitosan, dialdehyde starch	Schiff base reaction	NA	Eye drug delivery system	[48]
Sericin	Hydrogen bonding	100 kPa	Wound closure	[49]
Fibrin	Coagulation reaction	1.575 kPa	Corneal perforations	[50]
Poly(ethyleneglycol), peptide-based dendritic crosslinker	Pseudoproline/thiazolidine linkages	40–700 kPa	Corneal adhesive	[51]
Polydextran-aldehydes	Schiff base reaction	104.2 kPa	Wet adhesion, rapid hemostasis	[52]
Gelatin, tannic acid	Covalent bonding, hydrogen bonding, electrostatic interaction	42 J/m^2^	Wound closure	[46]
PVA, chitosan	Hydrogen bonding	15.3 kPa	Wound closure, self-healing	[53]
PEG	Hydrogen bonding	30 kPa	Prevent gas leakage	[54]
Pectin	Physical entanglement	3500 kPa	Wound closure	[55]
Polyethylene glycol diacrylate, dopamine	Amido link	21.6–656.7 kPa	Corneal perforations	[56]

## 3. Application of Adhesives in Corneal Repair

The eye, as an important organ for visual communication, has a complex structure and function. It consists of three essential parts: the eyeball, visual pathway, and accessory organs. Because the outermost barrier comes in contact with the external environment, the corneal epithelium is susceptible to damage, displacement, or burning from external stimuli.

**Figure 5:**
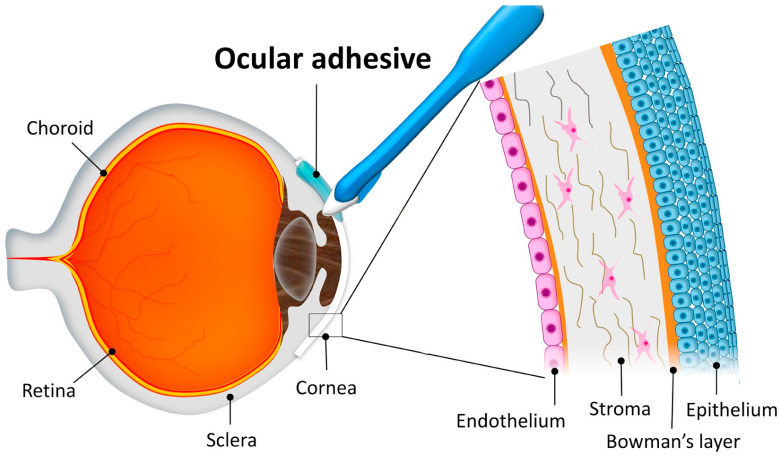
Corneal structure diagram. The outermost layer of the cornea is the corneal epithelium, followed by Bowman’s layer, the stroma, and finally, the endothelium (Reprinted with permission from Ref. [59]. 2019, ScienceDirect).

Unlike most organs, a healthy cornea is avascular, lacking blood and lymphatic vessels necessary for maintaining homeostasis and facilitating wound healing. Consequently, corneal wound healing is distinct. Unlike the skin, the cornea lacks an external barrier, making it relatively vulnerable. The cornea consists of three layers and two membranes (Figure 5): the outermost epithelial layer, which accounts for approximately 5% of the total corneal thickness, can regenerate and repair damage; the middle stroma makes up approximately 90% of the corneal thickness, serves as the primary refractive medium, and is composed of regularly arranged collagen fibers. Once the stromal layer is damaged, the corneal epithelium releases bioactive factors which induce corneal cells to transform into myofibroblasts. These myofibroblasts secrete type I collagen, creating scars in the cornea and affecting vision [57]. The final layer, i.e., the endothelial layer, consists of single flat hexagonal endothelial cells that cannot regenerate after damage and are maintained through the enlargement, expansion, migration, and sliding of adjacent cells. The anatomical structure of the cornea hinders the regeneration of stromal and endothelial cells after damage; thus, suitable materials are required to fill the wound and promote corneal repair.

Traditional suturing techniques can trigger inflammatory reactions in the corneal epithelial cells, leading to fluid leakage during repair. Moreover, the formation of new blood vessels and scars can affect the patient’s field of vision. With the development of laser technology, laser welding techniques have started being used in ophthalmic surgery; however, this method requires high technical expertise and has limited applicability [58]. In recent years, medical tissue adhesives have become a common method for postoperative closure. As mentioned earlier, fibrin-based tissue adhesive, cyanoacrylate tissue adhesive (CA), and others have been used for corneal perforation surgery since the 20th century. In addition to their common applications in corneal filling and tissue regeneration, medical tissue adhesives have been used for drug delivery, eye diagnostics, and biosensors.

### 3.1. Wound Sealant

Ocular tissue adhesives are primarily used for wound closure in the form of wound dressing, powders, or sealants. They create favorable conditions for wound healing after closing the wound area, preventing infection or other external damage [33]. To align with the normal physiology of the cornea, these adhesives must be transparent and possess strong mechanical properties. Additionally, antimicrobial agents or antioxidants are often incorporated to combat bacterial infections and oxidative stress while enhancing the biological functions of adhesives [60,61].

CA, a well-developed commercial glue, is the earliest adhesive used in clinical ophthalmic surgery. As an off-label product, CA can be used for various ophthalmic procedures, such as cataract repair, retinal detachment repair [62], scleral reinforcement [63], and corneal thinning repair [64]. However, due to its high cytotoxicity, its use in clinical ophthalmic surgery has gradually declined.

**Figure 6:**
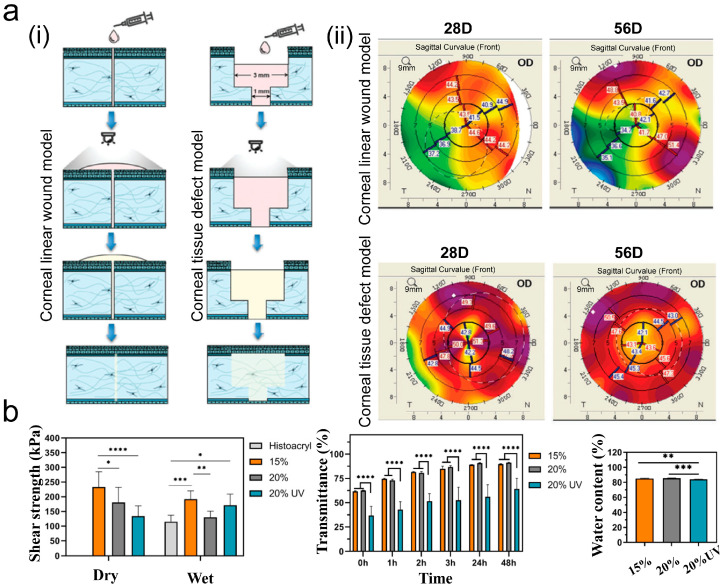
Use of photocrosslinked adhesives for wound closure. (**a**) Schematic diagram of corneal injury model (**i**) and corneal topography (**ii**) during postoperative corneal recovery (Reprinted with permission from Ref. [38]. 2023, Wiley); (**b**) shear strength, transmittance, and water content (Reprinted with permission from Ref. [66]. 2022, MDPI). Data are presented as mean value ± SD (* *p* < 0.05, ** *p* < 0.01, *** *p* < 0.001, and **** *p* < 0.0001).

Light can serve as an alternative to chemical crosslinking agents for connecting biomaterials and tissues. This approach is effective for modifying the mechanical and physical properties while enhancing biocompatibility [65]. Wang et al. [38] developed an injectable photocurable hydrogel based on hyaluronic acid (F20HD5). The viscous hydrogel effectively closed the penetrating linear corneal incisions and injuries, minimizing tissue loss in rabbits (Figure 6a). Figure 6a(ii) illustrates that the topographic map of corneal adhesion in the experimental group displayed moderate, symmetrical thickness changes after operation. Follow-up observations revealed that it promoted corneal repair, leading to more symmetrical curvature and fewer scars compared with the untreated control group. For deeply penetrating wounds involving corneal endothelial cells, Barroso et al. [66] developed a GelMA adhesive that can in situ crosslink within 2 min to fill the wound with a smooth surface. Its excellent mechanical properties, transmittance, and biocompatibility meet clinical demands (Figure 6b), making it a potential matrix filler or sealant for corneal and conjunctival applications.

**Figure 7:**
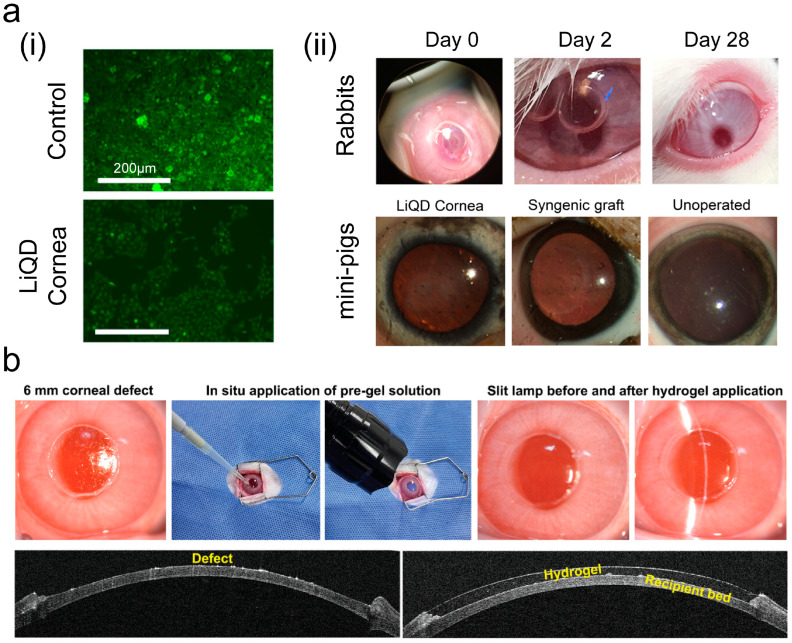
Example of adhesive used to close corneal perforation. (**a**) HCECs cultured on LiQD cornea and control plate (**i**). LiQD used in corneal perforation models of rabbits and pigs (**ii**) (Reprinted with permission from Ref. [69]. 2020, AAAS); and (**b**) corneal filling surgery in rabbits and anterior segment optical coherence tomography (AS-OCT) images of epithelial regeneration (Reprinted with permission from Ref. [70]. 2023, Wiley).

However, most patients with corneal perforation suffer from inflammation and photophobia, and the light curing method is not universal; therefore, some studies have cured the gel on the eye surface by controlling the temperature [67,68]. Griffith et al. [69] developed an injectable hydrogel matrix called “LiQD” with adhesive properties that can promote corneal defect filling in situ at body temperature and facilitate corneal regeneration. The application of the fibrin sealant effectively seals the cornea in situ during gelation, achieving a bursting pressure of 170 mmHg. This pressure is several times greater than the normal intraocular pressure range of 11–21 mmHg. The LiQD cornea demonstrated good biocompatibility, allowing human corneal endothelial cells (HCECs) to grow normally on the material (Figure 7a(i)). Its excellent sealing ability was confirmed in corneal perforation models involving rabbits and pigs (Figure 7a(ii)). The LiQD cornea not only conforms well to the corneal defect, successfully filling the wound, but also effectively promotes corneal cell growth and aids in injury repair.

Large-diameter corneal stroma defects represent a significant clinical challenge. Most tissue adhesives have poor adhesion performance and are only suitable for small-area injuries. Wang et al. [70] investigated a gelatin-based tissue adhesive capable of mimicking the human corneal microenvironment for repairing corneal stromal defects with a diameter of 6 mm (Figure 7b). This adhesive not only effectively and quickly closes wounds but also promotes the adhesion, proliferation, and repair of corneal cells.

Corneal wound healing differs from that of other tissues. To enhance the biocompatibility of materials, researchers often use similar substances to create adhesives. For example, You et al. [50] developed a biomaterial based on human platelet lysate which can improve adhesion and accelerate wound healing. In the application of ocular tissue adhesives, it is crucial not only to seal the wound but also to provide adequate burst pressure, transparency, and biocompatibility, as well as the ability to withstand prolonged cyclic deformation. Because blinking is an unavoidable physiological process, the adhesives must be able to overcome mechanical shear force and maintain performance stability. Therefore, the clinical use of ocular tissue adhesives requires more stringent conditions.

### 3.2. Drug Delivery

Eye injuries often require adjunctive surgical treatment with local drug delivery. Depending on the type of ocular surface disease, commonly used drugs include lubricants, antibacterial and anti-inflammatory drugs, growth factors, and intraocular drugs [71]. Conventional ocular drug delivery must overcome the barriers of the ocular surface, corneal epithelium, and blood–ocular barrier to achieve effective treatment [72] processes, such as tear flushing and blinking, which wash out liquid drugs easily from the cornea. Although ointments deliver drugs, they can obstruct vision. Consequently, the major challenges in corneal drug delivery lie in prolonging the residence time on the ocular surface while promoting corneal penetration.

**Figure 8:**
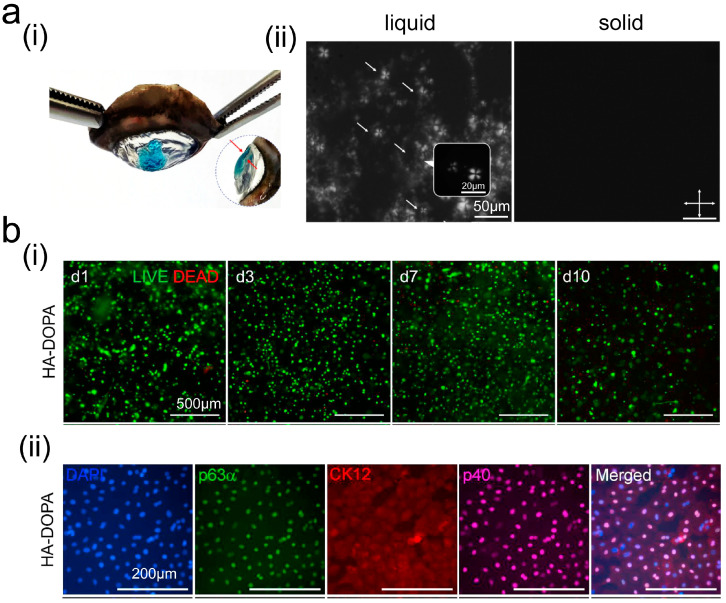
Physically connected bioadhesives and drugs. (**a**) Liquid crystal solidified on porcine cornea in vitro and red arrows indicate the thickness of t cubic phase (**i**). The characterization of liquid crystal adhesive loaded with acyclovir (white arrows) in different states by polarized light microscopy and crossed white double-arrows indicate the orientation of polarizers (**ii**) (Reprinted with permission from Ref. [75]. 2023, Nature); (**b**) hASC activity in adhesive (**i**) and expression of relevant markers (**ii**) (Reprinted with permission from Ref. [76]. 2019, ScienceDirect).

Common delivery systems consist of nanoparticles, immune cells, liposomes, and hydrogel carriers [73,74]. Most adhesive drugs are combined of a nanodelivery system and an adhesive. Nanodrug delivery systems can enhance the solubility of drugs, alter their distribution within the body, and improve drug targeting, thereby increasing therapeutic efficacy. A layered structure was developed using biological liquid crystals as an ophthalmic adhesive [75]. Due to the poor hydrophilicity of acyclovir, it was incorporated into the layered phase. The transition from liquid to solid was achieved through temperature changes, which enabled effective adhesion to the ocular surface and facilitated sustained drug release (Figure 8a). The layered phase containing acyclovir in Figure 8a(ii) exhibits a classic cross pattern, while the black image on the right confirms the presence of the cubic phase. In addition to conventional pharmaceuticals, active materials, such as cells, can be delivered. Koivusalo et al. [76] encapsulated human adipose-derived stem cells (hASCs) into a hyaluronic acid adhesive and connected corneal limbal epithelial stem cells (LESCs) through modification, thereby accelerating the regeneration of the corneal epithelium and repairing the corneal stroma. Figure 8b indicates that the cells in this adhesive are highly active and can express relevant markers, such as p63α and p40. However, the epithelial differentiation marker CK12 is expressed at low levels.

**Figure 9:**
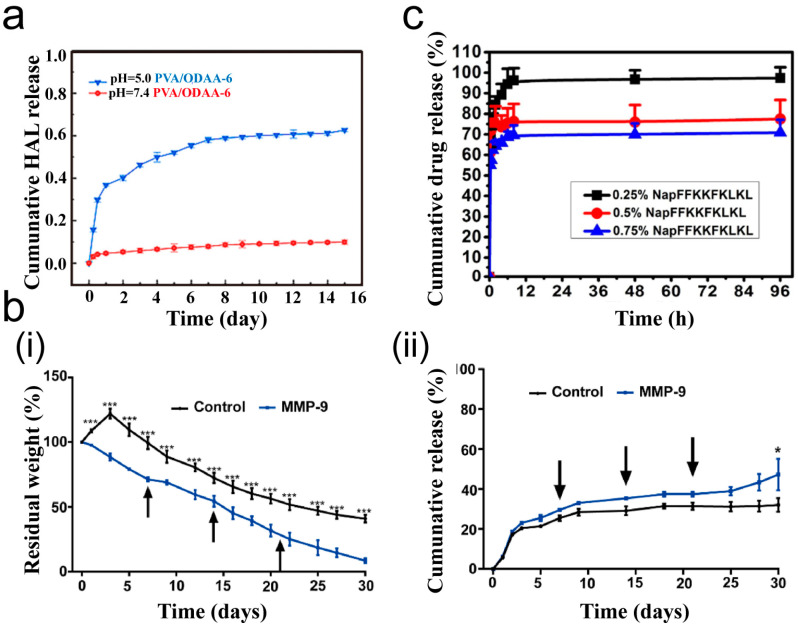
The sustained release effect of the hydrogel delivery system. (**a**) The sustained release time of aminolevulinic acid hexyl ester (HAL) loaded on gel at different pH values (Reprinted with permission from Ref. [78]. 2023, ScienceDirect); (**b**) the ratio of gel degradation (**i**) and drug release (*** *p* < 0.001) (**ii**) in different environments (* *p* < 0.05). The black arrow indicates the time point of adding MMP-9 again (Reprinted with permission from Ref. [79]. 2021, ScienceDirect); (**c**) drug release with different concentrations of polypeptide chains (Reprinted with permission from Ref. [80]. 2021, ScienceDirect).

Because of their high viscosity, adhesives present challenges in drug delivery. Several studies have used a hydrogel as a matrix carrier, incorporating drugs to facilitate sustained release [77]. Zhao et al. [78] loaded the antibiotic amikacin acid kanamycin onto ODex as a carrier, creating a pH-responsive hydrogel. Simultaneously providing multiple drugs to lesions in an optimized carrier with ideal dose ratios for specific therapeutic combinations has the most synergistic effects. Data have indicated that this hydrogel can continuously release drugs for up to 15 days in an acidic environment that simulates inflammation, demonstrating broad-spectrum antibacterial activity (Figure 9a). Drugs are linked to the adhesive through special interactions, and the sustained release time is often longer. Chen et al. [79] mixed hydrophobic rosiglitazone into a triglycerol monostearate solution. Due to the hydrophobic interactions, rosiglitazone was concentrated on the hydrophobic tail of triglycerol monostearate. As the triglycerol monostearate solution gelled in response to temperature, rosiglitazone was encapsulated in the gel and released during gel degradation. As shown in Figure 9b, its sustained release time was more than 30 days, which is longer than that shown in Figure 9a. Li et al. [80] reacted the amino group on the polypeptide with the phosphate group on dexamethasone sodium phosphate (Dexp) to form a self-assembled supramolecular hydrogel. The results show that the material released Dexp rapidly in the first 2 h and more steadily over the following 96 h (Figure 9c). Higher polypeptide concentrations enhanced the hydrogel’s mechanical strength, whereas ionic interactions within the gel network slowed the diffusion of Dexp, resulting in a slower drug release rate.

Drug delivery via contact lenses is a promising research area offering benefits such as extended wear, easy therapy termination, superior bioavailability compared with eye drops [81,82], and enhanced patient compliance [83,84].

### 3.3. Eye Diagnostics and Biosensors

Eyes contain crucial biological data related to human health that can be detected using biosensors. Advances in electronic technology, nanotechnology, biomaterials, and biosensors have led to an increase in the integration of sensors with biomaterials for clinical diagnosis [85,86]. Han et al. [87] used an ERG device with adhesive skin electrodes (RETeval system) to analyze the electroretinogram of the eyes of patients with vitreous hemorrhage. The data indicate that this detection method is effective in distinguishing between dense vitreous hemorrhage and posterior vitreous detachment in cases where it is difficult to differentiate these events from rhegmatogenous retinal detachment.

Intraocular pressure detection is a prominent area in ophthalmic monitoring. Shahbazi et al. [88] developed a chitosan- and indocyanine-based thin-film adhesive to seal penetrating corneal wounds. The adhesive’s ability to withstand pressure was evaluated, revealing that it sealed corneal injuries while enduring pressures of 140–320 mmHg—significantly exceeding normal intraocular pressure—and outperforming both Tisseel and traditional sutures. Chenault et al. [89] formulated an adhesive based on dextran aldehyde and ethylene glycol designed to seal leakages during corneal incisions in rabbits. A dosage of 1–2 μL with an IOP of at least 120 mmHg for up to 5 days demonstrated its efficacy in closing the wound while allowing for accurate IOP measurement under physiological conditions.

**Figure 10:**
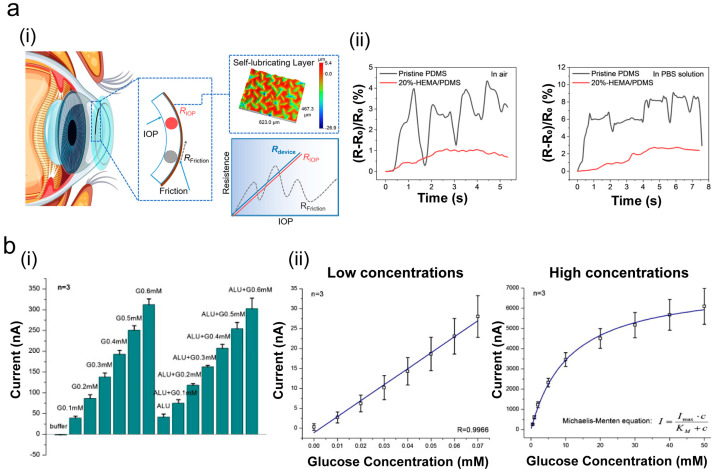
An artificial cornea used as a sensor. (**a**) A schematic of the corneal tonometer (**i**) and contact lens sensor showing a change in resistance upon application of friction in different environments (**ii**) (Reprinted with permission from Ref. [90]. 2023, ACS Publications); (**b**) repeatability and interference rejection (**i**): the test involving the use of three sensors which minimize ALU interference includes ascorbic acid and lactate urea. The calibration currents for low (0.01–0.07 mM) and high concentrations (0.5–50 mM) of glucose (**ii**) (Reprinted with permission from Ref. [91]. 2011, ScienceDirect).

Contact lenses are more common in biosensing detection than ophthalmic adhesives. Ren et al. [90] prepared a resistive–sensitive elastic film using silver nanoplates and a PEDOT–PSS mixture connected to a rigid ring with a gap to form a sensing layer during actual testing. The lubrication layer of the contact lens reduced the impact of the friction coefficients generated by blinking, enhancing the sensor’s sensitivity and linearity (Figure 10a). Applying lateral frictional force in the air/PBS solution, the 20%-HEMA/PDMS contact lens sensor showed a slighter resistance change compared to the control (PDMS). The test results on a rabbit high-intraocular-pressure model demonstrated that this sensor can safely and accurately detect eye pressure fluctuations.

In addition to intraocular pressure, tear component concentrations correlate with blood levels, allowing for the diagnosis of eye diseases by measuring cytokines and chemokines in tears [92,93]. Yao et al. [91] designed a contact lens with built-in sensors to monitor glucose levels in tears. These sensors effectively minimize interference from ascorbic acid, lactic acid, and urea in the tear film. The error margins detected by the three sensors are very small, even in the presence of these interferences (Figure 10b(i)). Moreover, this lens analyzes data within 20 s and detects glucose as low as 0.01 mM, showing high sensitivity (Figure 10b(ii)).

Adhesives can serve as connection sites between sensors and other tissues. Studies have explored the use of eye movement sensors to control wheelchair direction, providing significant benefits for disabled individuals [94]. Although ophthalmic sensors have significant diagnostic advantages, new challenges, such as those related to signal transduction, circuit design, biocompatibility, and sensitivity, have arisen during the fabrication process that must be urgently addressed.

The application of simple adhesives remains underexplored in the field of corneal detection and sensors, with most studies relying on the corneal lens. However, it is important to note that adhesives, as materials for sealing corneal wounds, are not confined to the ocular surface. They can penetrate deeply into defect sites and hold significant potential for various applications.

**Figure 11:**
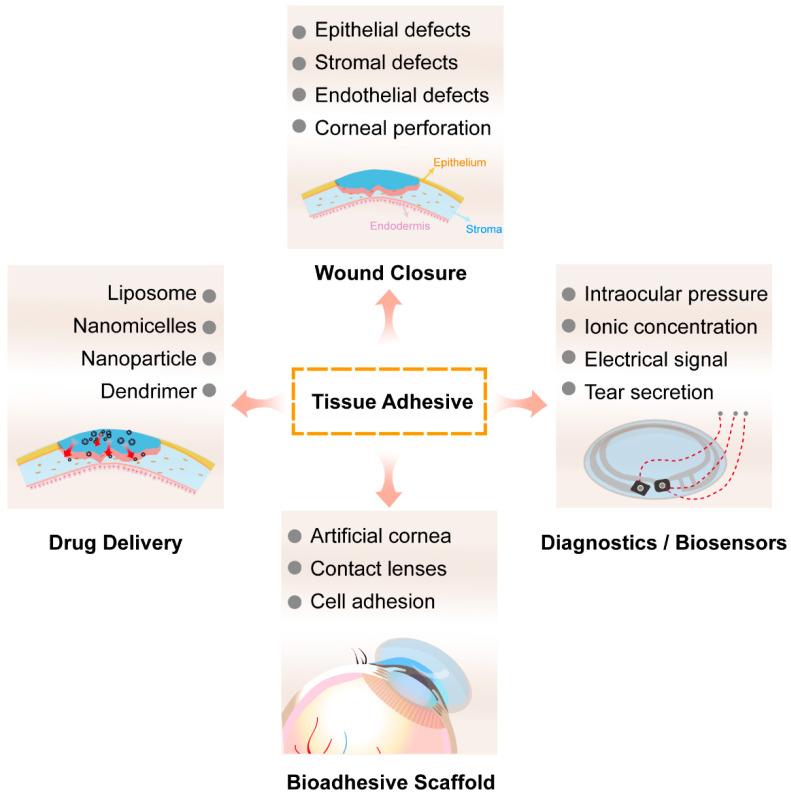
Schematic of applications of tissue adhesives.

Tissue adhesives are primarily used for wound closure, but they also have potential in drug delivery, biological scaffolds, and biosensor applications (Figure 11). While corneal adhesives currently have a single application, further development in various fields could enhance their functionality.

## 4. Summary and Outlook

The eye, an important means of communication with the external world, often experiences corneal damage due to external injuries or endogenous diseases. The unique physiological structure of the cornea necessitates more than just single-drug therapy. At present, corneal damage is treated using corneal transplant surgery, and maintaining corneal transparency post-surgery is a major challenge. Traditional sutures can lead to significant scar formation, an inability to effectively seal the cornea during recovery, and risks of liquid leakage and bacterial infection. Ocular medical tissue adhesives have emerged to address the shortcomings of surgical sutures. From the initial CA adhesive to safer fibrin-based adhesives, hyaluronic acid-based adhesives, and even multifunctional dendritic polymers, the selection of adhesives for ocular tissues has become more diverse. However, surgical suturing and even corneal transplantation remain primary clinical treatments, and tissue adhesives will take a long time to enter clinical application.

First, as medical devices for clinical use, the biosafety of tissue adhesives needs to be repeatedly verified. Most natural adhesives, such as fibrin and collagen, are extracted from animal tissues, which poses the risk of virus transmission. In addition, some patients may show immune reactions to these substances. Furthermore, the process from the early development of tissue adhesives to formal approval for clinical application is lengthy.

Second, the acceptance of tissue adhesives is not as high as that of surgical sutures. The public’s lack of trust in new medications implies that the process of understanding, accepting, and recognizing tissue adhesives will take time.

In addition, the simple usage of tissue adhesives and their low technical requirements for operators may undermine the interests of some healthcare professionals, leading to potential resistance from certain practitioners.

In future, the biosafety, bonding effectiveness, and multifunctionality of corneal adhesives should be investigated, integrating them with other disciplines for corneal closure, angiogenesis inhibition, wound healing promotion, and reactive oxygen species scavenging. In addition, the combination of corneal scaffolds, corneal contact lenses, and nanotechnology can aid in on-demand drug delivery, assistive therapies, and electronic sensor development for the real-time monitoring of healing. These advancements in the field of ophthalmology are expected to improve the clinical diagnosis of ophthalmic diseases and the effectiveness of various therapeutic approaches.

## Data Availability

The data that support the findings of this study are available upon reasonable request from the authors.
